# Determinants of satisfaction and self-perceived proficiency of trainees in surgical residency programs at a single institution

**DOI:** 10.1186/s12909-022-03521-5

**Published:** 2022-06-18

**Authors:** Segni Kejela, Abraham Genetu Tiruneh

**Affiliations:** College of Health Sciences, Addis Ababa University, Addis Ababa, Ethiopia

**Keywords:** Surgical resident satisfaction, Integrated sub-specialty residency training, Low resource country

## Abstract

**Background:**

We aimed to identify factors contributing to training program satisfaction and self-perceived proficiency of residents in 5 integrated surgical residency programs within the same referral institution.

**Methods:**

We conducted a cross-sectional survey including all senior surgical residents in all integrated sub-specialty and general surgery residency programs at Tikur Anbessa Specialized Hospital (TASH) in Addis Ababa, Ethiopia. Training programs were assessed on 6 educational components including operative case volume and diversity, intra-operative hands-on training, morning teaching sessions, seminars, ward rounds, and research opportunities.

**Results:**

Of 82 eligible residents, 69 (84.1%) responded to the survey. Overall resident satisfaction (rated from 0–10) varied between the 5 training programs, from a mean of 6.03 to 7.89 (overall *p* = 0.03). The percentage of residents who agreed they would be proficient by the end of their training ranged from 44.2%-88.9%. General surgery residents had the lowest overall satisfaction score, and lowest scores in all educational components except seminar teaching. In multivariable analysis, operative case volume and diversity (AOR 3.67; 95% CI, 1.24–10.83; *P* = 0.019), and hands-on training (AOR 4.15; 95% CI, 1.27–13.5; *P* = 0.018) were significantly associated with overall resident satisfaction. In ordinal logistic regression, hands-on training (OR 3.94, 95% CI, 1.69–9.2; *P* = 0.001), and seminar sessions (OR 2.43, 95% CI, 1.11–5.33; *P* = 0.028) were significantly associated with self-perceived proficiency.

**Conclusion:**

Different surgical residency training programs within the same institution had divergent resident satisfaction scores and proficiency scores. Operative case volume and diversity, and intraoperative hands-on training are the most important predictors of resident satisfaction while hands-on training and seminar sessions independently predicted self-perceived proficiency. Attention to these key components of resident education is likely to have a strong effect on training outcomes.

**Supplementary Information:**

The online version contains supplementary material available at 10.1186/s12909-022-03521-5.

## Introduction

In both medical and non-medical training programs, the satisfaction of trainees concerning the program conduct is associated with good outcomes. Surgical residency programs are not any different [1–4]. While results vary across training programs [1, 5–7], investigators concur that resident satisfaction is a key measure for assessing surgical residency program effectiveness [1]. In addition, acquiring a trainee’s input is associated with improvement in the learner’s well-being [6].

Surgical curricula utilize different educational methods, of which morning sessions, seminar sessions, intra-operative teaching, ward rounds, and research programs are the main ones [5–10]. Each component's contribution to the satisfaction of residents has been studied, but no study included all components, and none compared different residency programs [5–11]. We believe that each teaching method should be assessed individually, as the effectiveness and importance of the individual components can vary.

The main objectives of this study were first, to assess the satisfaction level of residents at one Ethiopian teaching institution with regards to each educational component of their training programs, and second, to compare levels of satisfaction and self-perceived proficiency between various sub-specialty integrated programs and the general surgery residency program at the same institution. By comparing satisfaction and efficacy scores between training programs at one institution we hoped to control for many factors that can vary between institutions, and identify key educational components that are strongly associated with resident satisfaction and proficiency.

## Methods

### Study design

This is a cross-sectional survey on satisfaction levels of residents in training across all sub-specialties within the Department of Surgery at Addis Ababa University.

### Study setting

Addis Ababa University is the one of the only two establishments in Ethiopia with surgical training in general Surgery, urology, pediatric Surgery, plastic, and reconstructive Surgery, and neurosurgery utilizing 7 affiliate hospitals and over 55 academic staff members consultant surgeons. Training in general surgery was started 41 years back, and the integrated sub-specialty residency programs were started 10 years ago. All hospitals involved in the training of all the programs are specialty level centers providing both emergency and elective surgical services.

### Study participants

Inclusion criteria for this study includes, all residents in their 3^rd^ to 5^th^ year of residencies were included. This included all residents enrolled in to, neurosurgery, general surgery, plastic and reconstructive surgery, pediatric surgery, and urology programs, provided that they have signed the informed consent. All residents enrolled in to this study had attached to their respective programs of study for at least 8 months.

General surgery residency is a 4 years training program, while the rest of the integrated sub-specialties trainings last for 5 years.

This study excluded, first and second year resident in any training program. The exclusion was intentional as all junior residents are enrolled in the same program until the end of the second year where they branch off into their respective training programs. In addition, residents opted out of the study were excluded.

### Study variables

The independent variables in this study were age, gender, field of specialty or sub-specialty of enrollment, sponsoring institution, and number of years in practice before joining residency.

The primary outcomes of this study were overall resident satisfaction with their residency training program (rated from 0 to 10), and self-perceived proficiency (rated on a 5-point Likert scale including strongly disagree, disagree, neutral, agree, and strongly agree). Satisfaction with 6 different educational components of the residency programs was also rated on a 5-point Likert scale ranging from very dissatisfied to very satisfied. The educational components were 1) operative case volume and diversity, 2) intraoperative hands-on training, 3) teaching in morning sessions, 4) seminar sessions, 5) ward rounds, and 6) research opportunities. In addition, regret in joining residency or surgery were evaluated. The survey instrument is shown in the Supplemental file.

Morning sessions are programs held at each unit on a biweekly to daily basis for the purpose of reporting activities during duty hours, discussions on challenging cases, and topics pertinent to the duty hour activities. Seminar sessions are fixed programs conducted throughout the year where the residents present the topic given, and consultants provide their perspectives and experiences. Ward rounds are held daily on patients with consultants leading the patient care, decision making, and teaching sessions simultaneously.

### Data source

A survey request forms were sent to all available residents using an online survey form and in-person paper format, based on whether the study participant was physically available or not. Each questionnaire was prepared with 3 categories. First category was basic information segment, followed by second part assessing resident satisfaction with regards to multiple teaching methods. Final category involved overall rating of the programs, and regret in joining surgery or the residency program. The questionnaire was initially validated using small pilot study before distribution to the participants.

### Measurement/analysis and interpretation

The collected data was coded, cleaned, and entered into SPSS statistics for Windows, version 23.0. Armonk, NY: IBM Corp, 2015. Descriptive statistics were done for categorical variables, and further analysis was done for difference in mean satisfaction and proficiency scores with analysis of variance and multivariable regression analysis.

### Bias

Social desirability bias was the expected during the data collection phase of this study. To alleviate this, data collection was done utilizing residents only as coordinators. Selection bias was the second bias expected from this study. This was reduced by enrolling as many eligible residents as possible maintaining anonymity.

### Study size

This is a study based on survey method of data collection. For this reason, data was collected from all residents that fulfilled the inclusion criteria, provided that they signed an informed consent.

### Statistical models

Simple frequency distribution was utilized to analyze gender, sponsoring institution, program of enrollment, year of residency, and regret in joining the program. Measures of central tendency with mean and measure of dispersion with standard deviation were used to evaluate the age of the respondents, number of years in practice before joining residency, and overall satisfaction of the residents with the program.

Satisfaction with the 6 components of residency education were first categorized into satisfactory and unsatisfactory with score 1–3 regarded as unsatisfactory and 4–5 as satisfactory, and simple frequency distribution was done. This was followed by measure of central tendency, mean, value determination for all the subcategories for each sub-specialty program. The mean values were compared using one-way analysis of variance (ANOVA) test, and a test of homogeneity (Levene’s test) was done, followed by Welch and Brown -Forsythe tests to determine *p*-values. Similarly, one-way ANOVA test was done for overall satisfaction scores of each program after calculating the mean values. In addition, univariable and multivariable regression analysis was done to evaluate association between the training components and the overall satisfaction of the trainees. Since the self-perceived proficiency was a Likert scale data, ordinal logistic regression analysis using the same independent variables as in the overall satisfaction was utilized.

### Ethical considerations

Ethical approval was acquired from the Institutional Review Board of Addis Ababa University. All participants gave their informed consent, and were informed their right to withdraw from the study at any time. Anonymity and confidentiality were assured throughout the data collection and analysis process. All data collected were treated confidentially and no individual except the authors had any access to any respondent information. The study was conducted in accordance with the Helsinki declarations, and institutional research ethics regulations of Addis Ababa University.

### Funding and conflicts of interest

No funding was acquired for this study. The authors of this study declare no financial and non-financial conflicts of interest.

## Results

Of 82 eligible residents, 69 completed the study survey (response rate of 84.1%). Participant demographics are shown in Table 1. Most participants were male. the mean age was 29.9 ± 1.5 years (range of 26–35 years). All respondents were in general practice before residency for a duration ranging from 6 months to 5 years, with an average duration of 1.8 ± 0.9 years. Participants’ fields of specialty are shown in Table 1. Only 4(5.8%) joined a surgical field that was not their first choice for residency training.

**Table 1:** Characteristics of the respondents with regards to sex, sponsor, specialty and year of residency

Variables	Category	Number	Percentage(%)
**Gender**	Male	60	87
	Female	9	13
**Sponsoring organization**	Academic institution	53	76.8
	Regional Hospitals	16	23.2
**Program of enrollment**	General Surgery	18	26.1
	Neurosurgery	17	24.6
	Plastic and reconstructive surgery	9	13
	Pediatric surgery	14	20.3
	Urology	11	15.9
**Year of residency**	3^rd^	27	39.1
	4^th^	21	30.4
	5^th^	21	30.4

**Fig. 1:**
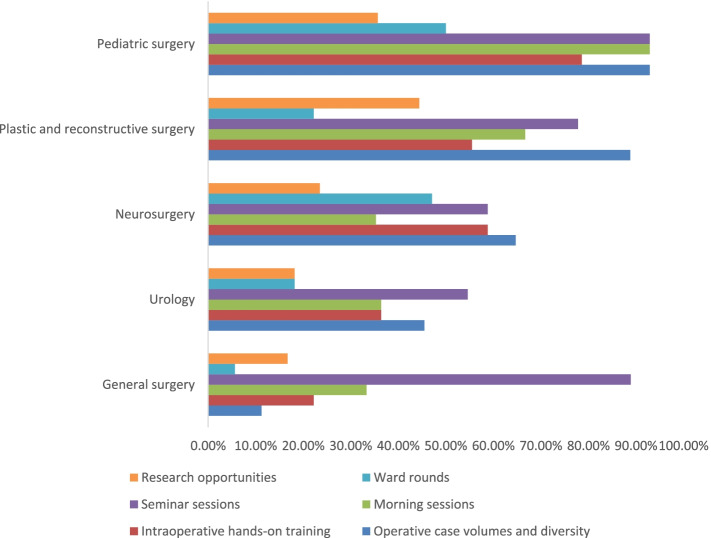
Satisfaction levels of a subgroup of residents with different categories of the training

Satisfaction with the educational components of training programs was assessed using 6 components including operative case volume and diversity, intra-operative hands-on training, morning teaching sessions, seminars, ward rounds, and research opportunities. Each component was assessed on a 5-point Likert scale ranging from very dissatisfied [1] to very satisfied [5]. Scores of 4 (satisfied) or 5 were regarded as satisfactory and very satisfactory respectively, and scores of 3 or less were regarded as unsatisfactory. Table 2 and Fig. 1 show satisfaction scores for the 6 educational components across the 5 surgical specialty residency programs.

**Table 2:** Subgroup of residents with satisfactory response (likert scale score of 4 and 5) with the different categories of the training

Categories	General surgery (*N* = 18)	Urology (*N* = 11)	Neurosurgery (*N* = 17)	PRS* (*N* = 9)	Pediatric surgery (*N* = 14)
Operative case volumes and diversity	2(11.2%)	5(45.5%)	11(64.7%)	8(88.8%)	13(92.9%)
Intraoperative hands-on training	4(22.2%)	4(36.4%)	10(58.8%)	5(55.5%)	11(78.6%)
Morning sessions	6(33.3%)	4(36.4%)	6(35.3%)	6(66.7%)	13(92.9%)
Seminar sessions	16(88.9%)	6(54.6%)	10(58.8%)	7(77.8%)	13(92.9%)
Ward rounds	1(5.6%)	2(18.2%)	8(47.1%)	2(22.2%)	7(50%)
Research opportunities	3(16.7%)	2(18.2%)	4(23.5%)	4(44.4%)	5(35.7%)

*PRS* Plastic and reconstructive surgery

General surgery and urology residents were least satisfied in most components of teaching. Pediatric surgery and plastic and reconstructive surgery residents had the highest satisfaction scores in most components studied. Notably, lowest satisfaction scores in operative case volume and diversity [2(11.2%)], and hands-on intraoperative training [4(22.2%)] was reported among general surgery residents. Highest satisfaction values in the same categories were seen in pediatric surgery, and plastic and reconstructive surgery with 13(92.9%) and 11(78.6%) respectively. Ward round and research related categories had lowest overall satisfaction scores across specialties. Seminar sessions had the highest overall satisfaction, with all residency programs reporting more than 50% favorable satisfaction scores.

Overall satisfaction score was rated using a scale of 0 to 10. As shown in Table 3, the lowest mean values were reported among general surgery and urology residents, with 6.03 ± 1.54 and 6.36 ± 2.06 respectively. The highest score was recorded among plastic and reconstructive surgery residents with a mean value of 7.89 ± 1.27.

**Table 3:** Mean values of overall satisfaction level of respondents with values reported from 0–10 for each respondent

Subgroups	Mean score (95%CI)	Standard deviation	Range
General surgery	6.03 (5.26–6.79)	1.54	4–9
Urology	6.36 (4.98–7.75)	2.06	2–9
Neurosurgery	7.18 (6.54–7.81)	1.24	5–9
Pediatric Surgery	7.75 (7.21–8.29)	0.94	6–9
PRS^*^	7.89 (6.91–8.86)	1.27	6–10

^*^Plastic and reconstructive surgery

Parametric statistics were performed for all the 6 components with a mean value of each. Test of homogeneity of variance showed 2 categories, satisfaction level with regards to ward rounds and satisfaction level with regards to seminar sessions, had a *p*-value of less than 0.05 and were excluded from the one-way ANOVA test. Further, a test of robustness, Welch and Brown-Forsythe tests, showed satisfaction with regards to research to have a *p*-value of > 0.05 and failed to accept the one-way ANOVA result. Finally, the one-way analysis of variance showed the means in 5 groups of residents were unequal in operative volume and diversity, and satisfaction with regards to hands-on training, F(4,64) = 7.51, *p* = 0.0001, and F(4,64) = 5.99, *p* = 0.002, respectively (Table 4). Similarly results from one-way ANOVA for the overall satisfaction indicated that the means of the resident groups were unequal, F(4,64) = 4.48, *p* = 0.03.

**Table 4:** One way ANOVA test for resident subgroups and categories of training satisfaction

Categories	Levene statistics (*p*-value)	Welch test (*p*-value)	Brown -Forsythe test (*p*-value)	F-test	*p*-value
Operative volume and diversity	0.289	0.001	< 0.001	7.51	< 0.001
Hands-on training	0.198	0.002	< 0.001	5.99	< 0.001
Morning sessions	0.041	< 0.001	0.002	4.98	0.001
Seminar sessions	0.029	0.017	0.007	4.46	0.003
Ward rounds	0.115	0.001	0.003	4.68	0.002
Research	0.668	0.325	0.283	1.41	0.242

As shown in Table 5, univariable logistic regression of all 6 components’ association with the overall satisfaction of the respondents showed statistically significant associations between satisfaction and both operative volume and diversity, and intraoperative hands-on training. Multivariable regression also showed satisfaction with regards to hands-on training is associated with a fourfold increase in overall satisfaction likelihood, and satisfaction with regards to the operative case volume and diversity was associated with 3.8-fold increase in overall satisfaction likelihood. Both outcomes were statistically significant.

**Table 5:** Univariable and multivariable logistic regression of overall satisfaction, with regards to satisfaction with all the teaching components

Categories	COR	*P*-value	AOR (95%CI)	*P*-value
Operative volume and diversity	3.78	0.014	3.67(1.24–10.83)	0.019
Hands-on training	4.0	0.018	4.15(1.27–13.5)	0.018
Morning session	1.07	0.87		
Seminar session	1.21	0.43		
Ward rounds	0.92	0.85		
Research	1.62	0.31		

*COR* Crude odds ratio, *AOR* Adjusted odds ratio, *CI* Confidence interval

All respondents were asked if they expect to be proficient at the end of their training. The highest reports of self-perceived proficiency were recorded among plastic and reconstructive surgery residents at 8(88.9%), neurosurgery residents at 14(82.3%), and pediatric surgery residents a 11(78.5%). On the other hand, urology and general surgery residents had the lowest self-perceived responses at 6(54.6%), and 8(44.2%) respectively. An ordinal logistic regression analysis was done to investigate effect of multiple variables on the self-perceived proficiency of the trainees. The predictor variables were tested a priori to verify there was no violation of the assumptions of no multicollinearity. The predictor variable, satisfaction with hands-on training, in the ordinal logistic regression analysis was found to contribute to the model. The ordered log-odds (Estimate) = 1.37, SE = 0.423, Wald = 10.51, *p* = 0.001. The estimated odds ratio favored a positive relationship of nearly fourfold [OR = 3.94, 95%CI (1.69,9.2)] compared to the reference variable. The predictor variable, satisfaction with seminar session similarly was found to contribute to the model. The ordered log-odds (Estimate) = 0.89, SE = 0.403, Wald = 4.85, *p* = 0.028. The estimated odds ration favored a positive relationship of nearly 2.5-fold [OR = 2.43, 95% CI (1.11,5.33)] compared to the reference variable. Satisfaction with operative case volume and diversity was found to trend towards statistical significance in its contribution to the model. The ordered log-odds (Estimate) = 0.68, SE = 0.374, Wald = 3.29, *p* = 0.07. The estimated odds ratio favored a positive relationship of nearly twofold [OR = 1.97, 95%CI (0.94,4.12)] compared to the reference variable.

Finally, regret in joining surgery was reported in 2(2.9%), and regret in joining the particular program of enrollment was 3(4.3%). Neurosurgery, General surgery, and Urology each had one respondent who regretted joining the program. No regret was reported in Plastic and reconstructive surgery, and Pediatric surgery.

## Discussion

In this study we found that resident satisfaction levels varied between surgical residency programs housed within the same teaching institution, and was associated with residents’ assessment of operative case volume and diversity, as well as the quality of their hands-on training experience. Resident self-perceived proficiency also varied between residency programs, and was associated with operative case volume and diversity as well as the quality of teaching seminar sessions. Odds ratios varied from 2.4 to 4.1, indicating a large effect size for these associations. Our findings suggest that particular components of surgical residency program curricula are key to resident satisfaction and self-perceived proficiency, and should be the focus of efforts to enhance the quality of surgical training.

Prior research has demonstrated fairly high satisfaction among general surgery residents with regards to their surgical training. For instance, a national survey from the US showed a satisfaction rate of 85% across the 248 surgical residency programs [12]. Other studies confirmed the same finding [6, 13, 14]. Similarly, studies on neurosurgery residents showed higher overall satisfaction score compared to our report [15, 16]. A study of pediatric surgery residents also found a high satisfaction rate [17]. On the other hand, satisfaction scores with regards to the training among urology residents are divergent across the literature, ranging from less than 30% to more than 80% [18, 19]. Similarly, publications show a varying level of satisfaction for plastic and reconstructive surgery residents across institutions of study [20, 21]. In general, satisfaction levels of trainees are institution and country dependent, and largely vary across specialties. Our finding of divergent satisfaction levels across different programs within an institution signals the necessity for every institution with surgical training programs to conduct their own assessment.

Hands-on intraoperative training and operative case volumes have been repeatedly sited across the literature as the most important predictors of satisfaction among surgical trainees. A national US survey of general surgery residents showed hands-on operative experience was a significant predictor of the overall satisfaction of the trainees [13]. Likewise, studies on plastic and reconstructive surgery residents showed operative experience as the most important element for the overall satisfaction of the respondents, with the volume and level of independent performance being the most important factors [21, 22]. Lack of operative exposure has been cited as a cause of burnout and dissatisfaction among neurosurgery residents in a US national survey, further signaling the importance of intra-operative training [16]. Our findings serve to reiterate that operative case volumes and hands-on training are crucial determinants of the overall satisfaction of the trainees, and should be a priority for surgical programs across institutions.

Self-perceived proficiency in surgical training is less well studied. In one large study, self-reported competency was associated with the overall satisfaction of residents [23]. A prior publication found only a poor association between operative case volume and self-reported proficiency [24]. To our knowledge, there is no publication correlating hands-on intraoperative experience and seminar sessions with self-perceived proficiency of surgical residents.

While case volume and diversity component was a determinant of both resident satisfaction and self-perceived proficiency, hands-on training was a determination of satisfaction only, and the quality of seminar teaching was a determination of proficiency only. These differences are interesting and somewhat unexpected, and suggest that cognitive training preferentially impacts self-perceived proficiency while hands-on procedural skill training preferentially imparts satisfaction. These findings merit further study.

To our knowledge, this is the first time integrated sub-specialty residency programs were compared with the general surgery residency program at the same institution. This approach kept many of the variables associated with residency training, such as infrastructure, salary, hospital culture and institutional educational policies constant between the training programs we analyzed. In addition, we analyzed various teaching components separately instead of making a collective assessment as was done in most studies referenced here, allowing us to identify particular educational elements that strongly contribute to resident satisfaction and self-perceived proficiency. Limitations of this study were the small sample size owing to small number of residents within each program, and the fact that this is a single institution study. We evaluated satisfactions of residents in all components using a self-reported likert scale survey. This may render the responses subjective as no logbooks or publications credentials were utilized to clearly evaluate the operative and research experiences of the respondents. The impact of work-life balance on the overall satisfaction of the residents has not been included in our study, and should be an area of research for the future.

## Conclusion

In this study, we found significant differences in the overall satisfaction, and satisfaction with regards to the teaching categories across different residency programs within the same institution. General surgery residents reported the lowest satisfaction rate in all except seminar teaching component while pediatric surgery residents had positive responses in almost all components, similar to plastic and reconstructive surgery. Operative case volume and diversity, and the hands-on training components of the training were significantly associated with the overall satisfaction. In addition, hands-on training and seminar sessions were significantly associated with self-perceived proficiency of the trainees. Attention to these key components of resident education is likely to have a strong effect on training outcomes.

## Supplementary Information

**Additional file 1.**

## Data Availability

The data and material for this study would be provided with a reasonable request to the corresponding author.
