# Supplementary material for: Determinants of satisfaction and self-perceived proficiency of trainees in surgical residency programs at a single institution
Source: BMC Med Educ. 2022 Jun 18;22:473. doi: 10.1186/s12909-022-03521-5 (PMC9206365; doi:10.1186/s12909-022-03521-5)
Supplement: Supplementary file 1 — Additional file 1. [file 12909_2022_3521_MOESM1_ESM.docx]

This questionnaire is intended to assess the level of satisfaction of residents regarding the training in surgery at Addis Ababa University College of Health Sciences Department of Surgery.

PI

**Part I:**

1. Age:________
2. Sex:
   1. M
   2. F
3. Year of residency:
   1. 3rd
   2. 4^th^
   3. 5^th^
4. Your sponsoring institution
5. Teaching institution/university
6. Regional health office
7. Self sponsor
8. Other
9. Number of years of service as a general practitioner before residency:________
10. Specialty:
    1. General surgerY
    2. Neurosurgery
    3. Plastic and reconstructive surgery
    4. Pediatric surgery
    5. Urology
11. Was the current specialty you are attending your first choice upon admission
    1. Yes
    2. No

**Part II:**

**Please rate your satisfaction with the following parameters from 1 - 5, [1: very dissatisfied, 5: very satisfied]**

|  |  | 1 | 2 | 3 | 4 | 5 |
| --- | --- | --- | --- | --- | --- | --- |
| 201 | Operative case volume and diversity |  |  |  |  |  |
| 202 | Satisfaction on intraoperative hands-on training provided |  |  |  |  |  |
| 203 | Satisfaction on teaching on Morning sessions |  |  |  |  |  |
| 204 | Satisfaction on teaching on seminar sessions |  |  |  |  |  |
| 205 | Satisfaction on teachings on Ward Rounds |  |  |  |  |  |
| 206 | Satisfaction on Research opportunities provided by the training program? |  |  |  |  |  |

**Part III**

301. I feel that I will be technically and scientifically proficient by the time I graduate from my training program

1. Strongly disagree;
2. Disagree;
3. Neutral;
4. Agree;
5. Strongly agree

302. What is your overall satisfaction rate with your current training program[rate it out of ten]____________________________________

303. Do you regret joining your sub-specialty?

1. Yes
2. No

304. Do you regret joining surgery?

1. Yes
2. No

305. What areas of improvement do you suggest on the following aspects of the training

1. Rounds
2. Seminars
3. Morning sessions
4. intraoperative teaching
5. Research
6. Workload
